# An analysis of the safety of Sevoflurane drugs: A disproportionality analysis based on Food and Drug Administration Adverse Event Reporting System

**DOI:** 10.1097/MD.0000000000038873

**Published:** 2024-08-30

**Authors:** Xinxia Yang, Yiming Shen, Hang Chen, Dongdong Chen

**Affiliations:** a Department of Anesthesiology, The Affiliated Lihuili Hospital, Ningbo University, Ningbo, Zhejiang Province, P. R. China; b Department of Otology and Skull Base Surgery, National Health Commission Key Laboratory of Hearing Medicine (Fudan University), Shanghai, P. R. China; c Department of Thoracic Surgery, Ningbo Medical Center Lihuili Hospital, Ningbo, Zhejiang Province, P. R. China.

**Keywords:** adverse events, atrial fibrillation, FAERS, Sevoflurane

## Abstract

Sevoflurane is a volatile anesthetic that can tolerate inhalation induction and is widely used for inducing anesthesia due to its pleasant odor. As a drug that has been on the market for nearly 30 years, the vast majority of adverse reactions have been documented. This study aims to improve the adverse reactions related to Sevoflurane through the mining, organizing and analysis of Food and Drug Administration Adverse Event Reporting System database data. We collected, organized, and analyzed reports from the first quarter of 2004 to the fourth quarter of 2022. We performed disproportionality analysis algorithms, including reporting odds ratio, the proportional reporting ratio values, to quantify the signal values of different adverse events (AEs). A total of 1126 AEs and 27 system organ classes were identified by performing statistics analysis system software. By combining algorithm calculations, we create a forest map of the top 30 AEs of the reporting odds ratio signal. Based on the reviewing relevant literature, we found that the vast majority of AEs have been reported in relevant studies. However, there is currently no study revealing the correlation between atrial fibrillation and Sevoflurane, which means that atrial fibrillation may be an unreported AE of Sevoflurane. In the present study, we found that atrial fibrillation may be a new adverse reaction of Sevoflurane through the Food and Drug Administration Adverse Event Reporting System database, which can function as a novel guideline to guide us in the more standardized use of Sevoflurane in clinical practice.

## 1. Introduction

Sevoflurane was first discovered by Pos Terell and successfully synthesized by Regan in 1968.^[1]^ However, it was not put into clinical use until phase III clinical trial from Japan in 1986, which was related to the potential AEs it may have.^[2]^ Sevoflurane is a colorless, transparent, aromatic, and nonvolatile liquid with stable chemical properties. At commonly used clinical concentrations, it will not burn or explode when in contact with oxygen.^[3]^ Due to its pleasant odor and nonirritating effect on trachea, Sevoflurane is permitted for inhalation induction in children and adults.^[4]^ In inhalation anesthesia, 95% to 98% of Sevoflurane is excreted through the lungs, while the rest is excreted through the liver and kidney pathways.^[5]^ Since its launch, there have been many reports of adverse events related to Sevoflurane, including neurological, respiratory, and circulatory systems. However, overall, Sevoflurane is considered a relatively safe and reliable drug.^[6]^ This study aims to explore the unreported adverse reactions of Sevoflurane through the FDA adverse event reporting system (FAERS) database.

Sevoflurane mainly acts on the human nervous system, which can increase dose-dependent intracranial pressure and reduce cerebrovascular resistance.^[7]^ However, there are also relevant studies reporting that Sevoflurane may damage the human nervous system. For instance, Jiang et al^[8]^ found that elderly patients over 65 years old are at increased risk of developing Alzheimer disease after inhaling inhaled anesthetics such as Sevoflurane. Moreover, Constant et al^[9]^ reported that Sevoflurane may be related to cortical epileptic electroencephalogram signs and usually has no clinical symptoms. Moreover, according to research conducted by Sun et al,^[10]^ we found an increased incidence of emergence delirium in children receiving Sevoflurane induced anesthesia. Malignant hyperthermia is a hereditary disease, which is characterized by hypermetabolic reaction to strong volatile anesthetic gases, including Halothane, Sevoflurane, etc.^[11]^ However, increasing evidence has demonstrated that Sevoflurane may induce hyperthermia malignant, postoperative restlessness, and agitation in children, which may be closely related to early pain.^[12,13]^ Zhao et al^[14]^ conducted a meta-analysis revealing that compared to propofol anesthesia, children who received Sevoflurane anesthesia had a higher risk of restlessness, postoperative nausea, vomiting, and postoperative pain. According to Kraus review,^[15]^ we found a significant increase in the incidence of diabetes insipidus in critically ill patients receiving Sevoflurane. Therefore, the incidence of neurological related adverse events (AEs) significantly increases in patients undergoing inhalation of Sevoflurane induced anesthesia.

In addition, Sevoflurane may also lead to complications in other systems. For example, similar to other halogenated volatile anesthetics, Sevoflurane can lead to dose-dependent cardiovascular suppression, which may affect blood flow in different organ systems.^[16]^ The Gonzalo Pascual team^[17]^ believes that the incidence of severe acute hepatitis significantly increases after receiving Sevoflurane anesthesia. Additionally, Goa et al^[18]^ proposed that similar to Halothane, Sevoflurane may cause emergency events such as cough, laryngospasm, agitation, and excitement, but Sevoflurane has a lower probability of causing arrhythmia than Halothane. Furthermore, Shutes et al^[19]^ found an unusual complication of Sevoflurane in severe childhood cases, which can cause hypercapnia. Besides, Barrons et al^[20]^ published a case report reporting on a 32 year old young woman who experienced rhabdomyolysis after receiving Sevoflurane.

In the present study, we obtained the AEs and clinical data of patients from the FAERS database, and then conducted disproportionality analysis to calculate the reporting odds ratios (RORs) values and corresponding 95% CIs of different system organ classes (SOCs) and PTs. Subsequently, we ranked the RORs values to obtain the AEs of Sevoflurane. Our research findings can help doctors better understand the risks associated with the use of Sevoflurane, thereby enabling more thorough communication with patients before surgery.

## 2. Materials and methods

### 2.1. Data acquisition

**Figure 1.**
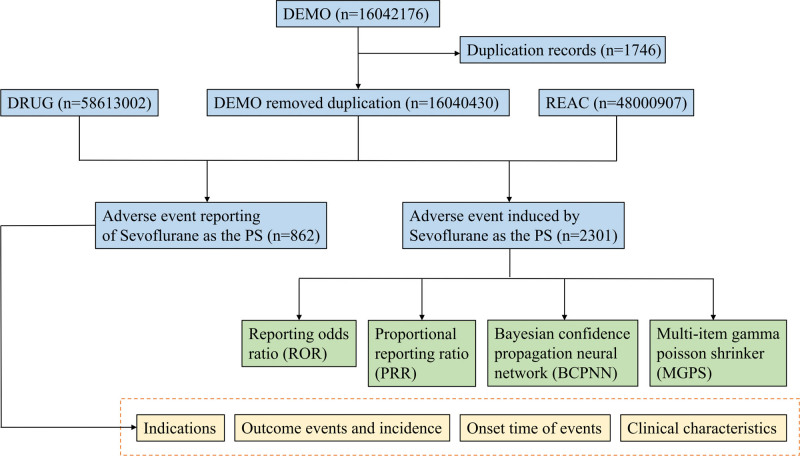
Flowchart for the safety analysis of Sevoflurane.

This study is a retrospective study aimed at exploring the adverse reactions of Sevoflurane through the FAERS database (https://fis.fda.gov/extensions/FPD-QDE-FAERS/FPD-QDE-FAERS.html). The AEs and clinical information of patients from over 100 countries were acquired from FAERS database. The FAERS database is a free and publicly available database that is updated quarterly and records adverse reactions from the first quarter of 2004 to the fourth quarter of 2022. Sevoflurane for inhalation was FDA certified and approved for sale in the United States in June 1995. Therefore, the AEs of Sevoflurane from the first quarter of 2004 to the fourth quarter of 2022 were included in our subsequent research (Fig. 1).

### 2.2. Data processing

The duplicate data were removed, and the obtained data were extracted and merged subsequently. To collect all the names of Sevoflurane (“Sevorane,” “Ultane,” and “BAX 3084”), we retrieved the Cochrane Library (https://www.cochranelibrary.com)^[21]^ and The Medical Subject Headings (MeSH, https://www.ncbi.nlm.nih.gov/).^[22]^ The Medical Dictionary for Regulatory Activities (MedDRA, version 26.0)^[23]^ were utilized for further supplementing the name of Sevoflurane.

### 2.3. Detection of drug signals

The disproportionality analysis was conducted to evaluate the relationship between the AE signals and Sevoflurane by performing the ROR and proportional reporting ratio (PRR) algorithm.^[24]^ The formula of ROR and PRR were listed as follows:


$$\begin{array}{l} ROR=\frac{\left(a/c\;\right)}{\left(b/d\;\right)} \end{array}$$

$$\begin{array}{l} PRR=\frac{a/\left(a+b\right)\;}{c/\left(c+d\right)\;} \end{array}$$

Equation: *a*, number of reports containing both the target drug and target adverse drug reaction; *b*, number of reports containing other adverse drug reaction of the target drug; *c*, number of reports containing the target adverse drug reaction of other drugs; *d*, number of reports containing other drugs and other adverse drug reactions.

### 2.4. Signal analysis

Based on the AE signals, we explored the potential relationship among the AE signals, SOC and preferred term (PT). We recognized the clinical information of patients (e.g., survival outcomes, reporter, age, and reporting country), which was shown in Table 1. The forest map was generated to exhibit the RORs and 95% CI of AEs related to Sevoflurane.

**Table 1:** The survival outcomes, reporter, age, and reporting country of AE patients.

	Female	%	Male	%
Outcome				
Death	71	6.81	108	9.77
Hospitalization	253	24.28	251	22.71
Life-threatening	146	14.01	173	15.66
Disability	19	1.82	31	2.81
Other serious	520	49.90	504	45.61
Required intervention	26	2.50	32	2.90
Not specified	7	0.67	6	0.54
Reporter				
Healthcare professional	694	84.94	705	84.74
Consumer	29	3.55	25	3.00
Not specified	94	11.51	102	12.26
Age				
<18	177	21.66	244	29.33
18 to 64	327	40.02	309	37.14
≥65	125	15.30	111	13.34
Not specified	188	23.01	168	20.19
Reporting countries				
United States	69	8.45	244	22.26
Other country	44	5.39	836	76.28
Not specified	704	86.17	16	1.46

## 3. Results

**Figure 2.**
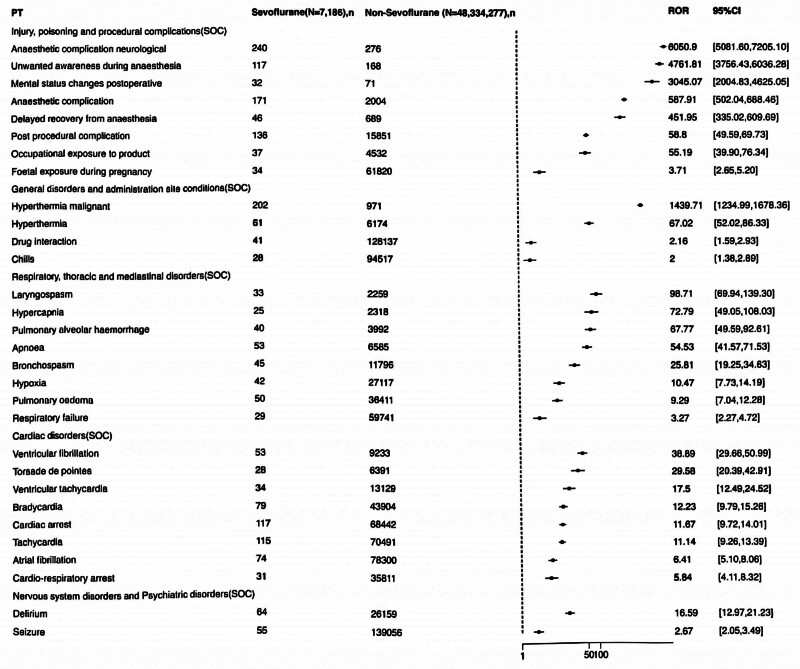
Identification of the AE signals related to Sevoflurane. We filtered 1126 AE signals related to Sevoflurane, 558 of which were identified as positive signals (as shown in this figure). Regarding AE signals, we obtained 5 SOCs (injury, poisoning, and procedural complications, general disorders and administration site conditions, respiratory, thoracic and mediastinal disorders, cardiac disorders, and nervous system disorders and psychiatric disorders), in which injury, poisoning, and procedural complications exhibited the strongest signals. AE = adverse event, SOCs = system organ classes.

**Figure 3.**
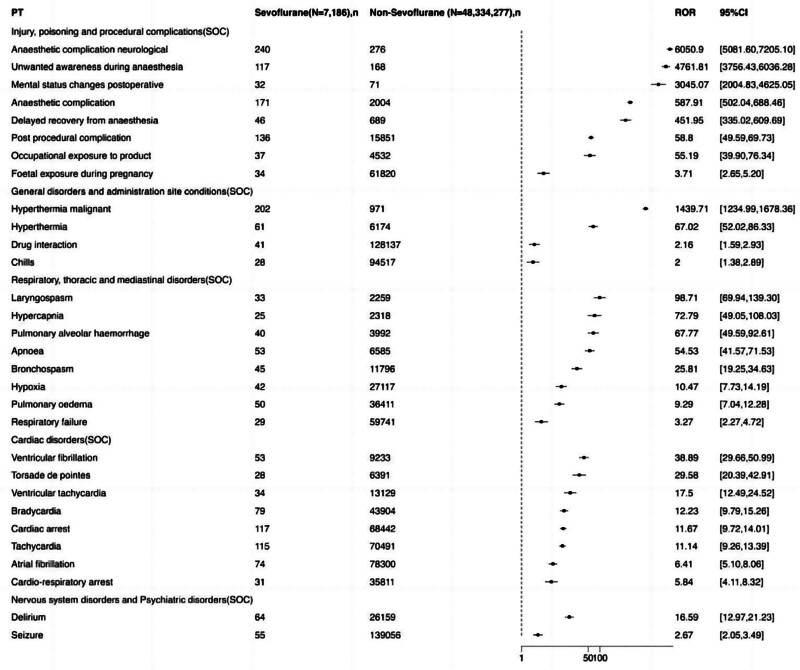
Time to onset of sevoflurane-associated adverse events. The vast majority of AEs occurred within 30 days after receiving Sevoflurane anesthesia. AEs = adverse events.

Data extraction obtained 1649 Sevoflurane AE reports, 817 women and 832 men. The survival outcomes, reporter, age, and reporting country of AE patients are shown in (Table 1). Men reported more ADEs, and the reported clinical outcomes of ADEs were more serious. We filtered 1126 AE signals related to Sevoflurane, 558 of which were identified as positive signals (Fig. 2). Regarding AE signals, we obtained 5 SOCs (injury, poisoning and procedural complications, general disorders and administration site conditions, respiratory, thoracic and mediastinal disorders, cardiac disorders, and nervous system disorders and psychiatric disorders), in which injury, poisoning and procedural complications exhibited the strongest signals (Table 2). Additionally, it is worth mentioning that according to time to onset of Sevoflurane-associated AEs in Figure 3, we found that the vast majority of AEs occurred within 30 days after receiving Sevoflurane anesthesia.

**Table 2:** Signal strength of reports of Sevoflurane at the Top 30 PTs in FAERS database.

PT	Sevoflurane (N = 7186), n	Non-Sevoflurane (N = 48,334,277), n	ROR	RORL	RORU	PRR	χ^2^	EBGM	EBGM05	IC2	ICO25
Injury, poisoning, and procedural complications (SOC)											
Anaesthetic complication neurological	240	276	6050.902114	5081.599149	7205.097318	5848.845823	750579.8696	3128.917533	2703.666523	11.61144792	9.940394323
Unwanted awareness during anesthesia	117	168	4761.812772	3756.431582	6036.276817	4684.298842	322932.6767	2761.681423	2264.605489	11.43133119	9.75639037
Mental status changes postoperative	32	71	3045.070013	2004.830766	4625.054415	3031.514455	66825.94424	2089.995401	1473.199672	11.02928405	9.339204364
Anaesthetic complication	171	2004	587.9060612	502.0372255	688.4619691	573.9398858	90116.81085	528.8949569	463.4392795	9.046837409	7.379461418
Delayed recovery from anesthesia	46	689	451.9490042	335.0206032	609.6875846	449.0623281	19278.20332	421.0203321	327.72726	8.717746096	7.048126837
Post-procedural complication	136	15,851	58.80386933	49.59117096	69.7280379	57.70989129	7516.914698	57.22746524	49.62320993	5.838635802	4.17225131
Occupational exposure to product	37	4532	55.19262241	39.90354231	76.33972805	54.91358998	1942.800393	54.47699492	41.52815901	5.767575218	4.100800374
Foetal exposure during pregnancy	34	61,820	3.712118358	2.650041008	5.199852629	3.699286181	67.01563016	3.697802433	2.78914761	1.886668146	0.220399532
General disorders and administration site conditions (SOC)											
Hyperthermia malignant	202	971	1439.707976	1234.991379	1678.35913	1399.265587	233648.1306	1158.473048	1018.944737	10.17800876	8.509653821
Hyperthermia	61	6174	67.01593628	52.02241074	86.33078806	66.45554495	3894.732931	65.81516191	53.24704363	6.040348072	4.373719241
Drug interaction	41	128,137	2.158785187	1.588069505	2.93460297	2.152173694	25.34875163	2.15180515	1.664304559	1.105547445	-0.560700267
Chills	28	94,517	1.996464293	1.377394907	2.893774075	1.992581605	13.86742849	1.992287646	1.460386592	0.994425959	-0.671828688
Respiratory, thoracic, and mediastinal disorders (SOC)											
Laryngospasm	33	2259	98.7061954	69.94378565	139.2963352	98.25750288	3131.242159	96.85719852	72.60396592	6.59778737	4.930469923
Hypercapnia	25	2318	72.79261173	49.04931031	108.0293339	72.54284617	1745.179239	71.7794782	51.58678409	6.16549953	4.498205774
Pulmonary alveolar hemorrhage	40	3992	67.76817699	49.58948363	92.61088191	67.39652001	2590.713498	66.73782437	51.39022466	6.060432751	4.39358514
Apnoea	53	6585	54.53105766	41.57370577	71.52685081	54.1362419	2742.506128	53.71198447	42.80384471	5.74717212	4.080567603
Bronchospasm	45	11,796	25.81475103	19.24578725	34.62583068	25.65935668	1062.632291	25.56564237	19.9961342	4.676134371	3.009695172
Hypoxia	42	27,117	10.4731593	7.73116746	14.18764582	10.41779154	357.2265277	10.40322741	8.069732853	3.378959263	1.712639049
Pulmonary edema	50	36,411	9.294167574	7.036015871	12.27705458	9.236456973	367.0093911	9.225162087	7.308469345	3.20557426	1.539277514
Respiratory failure	29	59,741	3.274255765	2.273459852	4.715610354	3.265077722	45.60340001	3.263978722	2.405400319	1.706631652	0.04036163
Cardiac disorders (SOC)											
Ventricular fibrillation	53	9233	38.88956346	29.65800111	50.99460818	38.61011079	1930.997164	38.39545046	30.60578086	5.262863469	3.596366233
Torsade de pointes	28	6391	29.57980577	20.39222929	42.90678068	29.46844555	766.8093954	29.34426476	21.4963619	4.875006656	3.208389305
Ventricular tachycardia	34	13,129	17.49673469	12.48646126	24.51741277	17.41868167	524.9709095	17.37627226	13.10268277	4.119046708	2.452628694
Bradycardia	79	43,904	12.22636188	9.792899488	15.26452151	12.10294376	803.9460687	12.08300123	10.03518428	3.594906937	1.92862191
Cardiac arrest	117	68,442	11.67199283	9.721529568	14.01378411	11.49823508	1121.14516	11.48031922	9.851639664	3.521090853	1.85482619
Tachycardia	115	70,491	11.13537169	9.260216501	13.39023799	10.9731719	1042.219298	10.95692803	9.390313702	3.453771464	1.787507857
Atrial fibrillation	74	78,300	6.41252871	5.099378983	8.063829851	6.356791565	334.270206	6.351733733	5.243570526	2.667150435	1.000890336
Cardio-respiratory arrest	31	35,811	5.843444783	4.105716195	8.316660311	5.822550434	123.8078906	5.818379376	4.330592561	2.540617368	0.874319439
Nervous system disorders and psychiatric disorders (SOC)											
Delirium	64	26,159	16.59498963	12.97084464	21.23174614	16.45609743	927.3148803	16.41837519	13.35956326	4.037239456	2.370915959
Seizure	55	139,056	2.673169495	2.050141986	3.485531832	2.660363439	57.1357407	2.659706985	2.130131175	1.411267315	-0.254978865

The injury, poisoning, and procedural complications included anesthetic complication neurological, unwanted awareness during anesthesia, mental status changes postoperative, anesthetic complication, delayed recovery from anesthesia, post-procedural complication, occupational exposure to product, and fetal exposure during pregnancy. General disorders and administration site conditions exhibited the second strongest signal strength, including hyperthermia malignant, hyperthermia, drug interaction, and chills. Respiratory, thoracic and mediastinal disorders showed the third strongest signal strength, including laryngospasm, hypercapnia, pulmonary alveolar hemorrhage, apnoea, bronchospasm, hypoxia, pulmonary edema, and respiratory failure. Cardiac disorders emerged as the fourth strongest signal strength, including ventricular fibrillation, torsade de pointes ventricular tachycardia, bradycardia, cardiac arrest, tachycardia, atrial fibrillation, and cardio-respiratory arrest. Nervous system disorders and psychiatric disorders revealed the fifth strongest signal strength, including delirium and seizure.

## 4. Discussion

According to the calculation of adverse reaction signals of Sevoflurane in the FAERS database, we found that most AEs have been reported. For instance, as stated in the introduction, the main AE of Sevoflurane is its neurotoxicity, which can also cause epilepsy, delirium, postoperative restlessness, and agitation. This means that it undeniably causes anesthetic complication neurological, unwanted awareness during anesthesia, and mental status changes postoperative.^[25–27]^ Furthermore, as an inhaled anesthetic, Sevoflurane inevitably causes anesthesia complications (postoperative nausea, vomiting, postoperative pain), Delayed recovery from anesthesia, post-procedural complication, and occupational exposure to product. As for fetal exposure during pregnancy, increasing evidence have demonstrated that the fetal toxicity caused by exposure to Sevoflurane during pregnancy involves oxidative stress, neuroinflammation, and cognitive impairment.^[10]^

As reported, Sevoflurane can cause hyperthermia malignant, hyperthermia, and drug interaction. According to relevant research, children receive Sevoflurane induced anesthesia are often more prone to hyperthermia malignant and hyperthermia, which is related to the damage of thermoregulation center.^[28]^ Additionally, Sevoflurane can generate additive interactions with N(2)O and synergistic interactions with opioid drugs.^[29]^

As for the complications of the respiratory, thermal, and medical disorders system, they are consistent with the reported findings as well. For instance, after receiving Sevoflurane inhalation anesthesia, the child can cause laryngospasm and can be treated with intramuscular injection of succinylcholine.^[30]^ Furthermore, Brittany team has reported that Sevoflurane may cause hypercapnia in children.^[19]^ Ahmed-Khan et al^[31]^ reported that Sevoflurane could cause diffuse pulmonary alveolar hemorrhage. Therefore, if hemoptysis and hypoxemia occur after Sevoflurane induction, we should be alert to the possibility of alveolar hemorrhage. In addition, the recent literature have also reported that Sevoflurane can cause hypoxia, apnea, hypoxemia, bronchial obstruction, and pneumothorax.^[32]^ According to a case report from Japan, we found that a 9-year-old Japanese boy developed bronchospasm and hypercapnia after Sevoflurane anesthesia.^[33]^ Moreover, related literature have reported that Sevoflurane can cause pulmonary edema and airway bleeding in goats, and can also cause pulmonary edema in humans.^[34,35]^ In addition, according to a retrospective study of 5864 cases conducted in Spain, we found that although Sevoflurane could have good anesthesia effects, it had also led to severe respiratory failure in several cases.^[36]^

Regarding cardiac disorders, we found that the vast majority of AEs were the same as reported. For example, under Sevoflurane anesthesia, it is more likely to cause ventricular fibrillation and torsade de pointe.^[37,38]^ Besides, Sevoflurane can cause multiform ventricular tachycardia, which is believed to be caused by prolonging the Q-T interval.^[39]^ Furthermore, related studies have also found that children experience significant rapid onset of bradycardia during Sevoflurane induced anesthesia.^[40]^ Furthermore, related cases have also been reported to experience sudden cardiac arrest after receiving Sevoflurane anesthesia.^[41]^

However, there are also several AEs have not been reported yet, which are also the AE need to be focused on. At present, there are no relevant research reports on the correlation between atrial fibrillation and Sevoflurane. However, based on our ROR values, we found that there is a certain possibility of inducing atrial fibrillation in patients receiving Sevoflurane anesthesia. Therefore, we do not recommend using Sevoflurane anesthesia for patients with arrhythmia.

We were the first to conduct a systematic analysis of adverse reactions of Sevoflurane drugs through the FAERS database. In addition, based on our results, we found that atrial fibrillation is also a common AE of Sevoflurane, but there are currently no relevant reports on the correlation between the 2. However, our research is still remain an analysis of public database, and the correlation between atrial fibrillation and Sevoflurane needs further clinical research to confirm.

In Summary, we downloaded and analyzed the results of the FAERS database, and obtained the most common adverse reactions of Sevoflurane, most of which are closely related to clinically discovered adverse reactions. In addition, we also found that Sevoflurane can cause a novel AE, namely atrial fibrillation, which provides a theoretical basis for guiding our clinical use of Sevoflurane.

## Acknowledgments

We thank the FAERS database for generously sharing a large amount of data.

## Author contributions

**Writing – original draft:** Xinxia Yang.

**Writing – review & editing:** Xinxia Yang, Dongdong Chen.

**Formal analysis:** Yiming Shen.

**Data curation:** Hang Chen.

**Investigation:** Hang Chen.

**Software:** Dongdong Chen.

**Supervision:** Dongdong Chen.
